# Application of ^1^H-NMR Metabolomic Profiling for Reef-Building Corals

**DOI:** 10.1371/journal.pone.0111274

**Published:** 2014-10-29

**Authors:** Emilia M. Sogin, Paul Anderson, Philip Williams, Chii-Shiarng Chen, Ruth D. Gates

**Affiliations:** 1 Hawaii Institute of Marine Biology, Kaneohe, Hawaii, United States of America; 2 University of Hawaii at Manoa, Honolulu, Hawaii, United States of America; 3 College of Charleston, Charleston, South Carolina, United States of America; 4 National Museum for Marine Biology and Aquarium, Checheng, Taiwan; National Research Council of Italy, Italy

## Abstract

In light of global reef decline new methods to accurately, cheaply, and quickly evaluate coral metabolic states are needed to assess reef health. Metabolomic profiling can describe the response of individuals to disturbance (i.e., shifts in environmental conditions) across biological models and is a powerful approach for characterizing and comparing coral metabolism. For the first time, we assess the utility of a proton-nuclear magnetic resonance spectroscopy (^1^H-NMR)-based metabolomics approach in characterizing coral metabolite profiles by 1) investigating technical, intra-, and inter-sample variation, 2) evaluating the ability to recover targeted metabolite spikes, and 3) assessing the potential for this method to differentiate among coral species. Our results indicate ^1^H-NMR profiling of *Porites compressa* corals is highly reproducible and exhibits low levels of variability within and among colonies. The spiking experiments validate the sensitivity of our methods and showcase the capacity of orthogonal partial least squares discriminate analysis (OPLS-DA) to distinguish between profiles spiked with varying metabolite concentrations (0 mM, 0.1 mM, and 10 mM). Finally, ^1^H-NMR metabolomics coupled with OPLS-DA, revealed species-specific patterns in metabolite profiles among four reef-building corals (*Pocillopora damicornis, Porites lobata, Montipora aequituberculata,* and *Seriatopora hystrix*). Collectively, these data indicate that ^1^H-NMR metabolomic techniques can profile reef-building coral metabolomes and have the potential to provide an integrated picture of the coral phenotype in response to environmental change.

## Introduction

Coral reefs are among the most productive ecosystems in the world [1]. Globally, they produce up to 700×10^12^ g C year^−1^ [2] and provide important services (e.g., fisheries habitat, coastal protection, and promotion of tourism) that support tropical and subtropical coastal communities worldwide [3]. Despite the importance of coral reefs, damaging anthropogenic activities such as overfishing, pollution, and physical destruction jeopardize their long-term persistence [4]–[6]. Of particular concern are recent increases in sea surface temperatures and ocean acidification that are driving worldwide declines in coral reef ecosystems [7], [8].

Coral susceptibility to environmental stress varies within and among species [9], [10]. This feature reflects the combined physiology of a diverse assemblage of microorganisms and algal partners that comprise the coral holobiont [11]–[15]. While research has focused on describing differential responses of corals and their partners to environmental stress [12], [13], [16]–[19], the development of tools that can be broadly deployed and used to rapidly assess coral health trajectories is still in its infancy. Applications of ‘omics’ techniques are enhancing these efforts and are describing coral resistance, resilience, and function [20]. While there are challenges and limitations in interpreting these large datasets [20], global analyses of genes [21], transcripts [22], and proteins [23] are advancing our understanding of holobiont responses to ecological disturbances. However, these techniques are currently too expensive to apply in reef-wide monitoring programs. Consequently, there is still a need for cost-effective molecular tools that can be proactively used to assess coral metabolic states.

Metabolites are small molecules that are products and intermediates of metabolism and play essential roles in biochemical pathways that underpin growth, nutrition, reproduction, and survival. As such, external disturbances can trigger responses in the metabolic processes of coral colonies leading to shifts in metabolite profiles. Despite their broad significance in maintaining basic biological functions, metabolite research is far from complete in reef corals. For instance, select groups of compounds, such as sugars, lipids and mycosporine like amino acids have received considerable attention in the literature due to their biochemical roles in maintaining coral metabolism. However, other metabolites such as steroids, isoprenoids, alkaloids, and sulfur containing compounds, such as dimethylsulphoniopropionate and acrylate, are also critical for coral reproduction, growth, survival and maintenance of symbiotic status [24]–[27]. A more comprehensive description of coral metabolite composition could further elucidate the role of metabolites and pinpoint metabolic pathways essential for coral resilience to environmental change, thereby providing a means to gauge coral biological performance.

Advances in metabolomic technologies provide an opportunity to quantify many metabolites simultaneously. This holistic approach takes advantage of metabolite profiling methods using nuclear magnetic resonance (NMR) and/or mass spectrometry (MS) techniques to capture organism responses to external conditions. Changes in the metabolome typically reflect gene and protein expression [27]. Thus, metabolomics can describe and integrate complex responses of organisms. These methods are applicable across scientific disciplines and can identify bioactive compounds, assess food safety, and describe the function of unknown genes [28]–[30]. Of particular interest to coral reef scientists is the capacity of metabolomic tools to identify metabolites and profiles that may serve as biomarkers for disease or stress response [31]–[34]. These techniques have only recently been applied to corals [35], [36] and extensions of these methods may enable rapid and cost-effective assessment of coral metabolic states.

Here, we demonstrate the application of metabolomic profiling in reef-building corals using proton-NMR (^1^H-NMR) spectroscopy. ^1^H-NMR metabolomic methods are tractable because they are relatively inexpensive (typically<$5/sample), reproducible, require minimal sample preparation, are non-destructive allowing for repeat analyses using various acquisition experiments, and can be used in a non-targeted approach to measure multiple metabolite classes in a single run [37]. Furthermore, ^1^H-NMR techniques have been used to quantitatively investigate the concentration of select metabolites in *Acropora spp.* corals [38], [39]. To determine the efficacy of profiling reef-building coral metabolomes using ^1^H-NMR methods, we conducted three independent experiments to assess the variability, sensitivity, and ecological relevance of our methods. First, we investigated variation in ^1^H-NMR profiles within and among multiple coral colonies. Then, we explored the sensitivity of our techniques by spiking samples with various concentrations of metabolites known to occur in reef-building corals. Finally, we compared metabolite profiles from four coral species to determine if our methods describe signatures inherent to taxonomic divisions. Our results collectively demonstrate that ^1^H-NMR techniques are a viable and powerful tool for assessing the metabolomes of reef-building corals.

## Methods

Corals sampled in Hawaii were collected under special activity permits issued by the Department of Land and Natural Resources (permit numbers 2011-1, 2012-63) to the Hawaii Institute of Marine Biology (HIMB). Corals in Taiwan were collected under a research permit issued to the National Museum for Marine Biology and Aquarium (NMMBA) from the Kenting National Park of Taiwan.

### Coral Sampling and Metabolite Extractions

#### Technical, Intra-, and Inter-Colony Variability

Reef-building coral samples were collected from a small area (ca. 135 m^2^) of a fringing reef in Kaneohe Bay, Hawaii (21°25′58.28″N, 157°47′23.55″W) using bone cutters and immediately immersed in liquid nitrogen. Samples used to assess intra-colony and technical variation in metabolite profiles were collected in December 2011, while those used to assess inter-colony variability were sampled in June 2013. While coral metabolite content may change, variability measured in ^1^H-NMR profiles is still likely to be comparable within and among colonies through time. Following sample collection, coral fragments were lyophilized and stored at −80°C prior to metabolite extraction. To assess technical variation, three replicate fragments from separate colonies were collected, pooled, and pulverized. From these samples, 5 replicate extractions were performed. Five fragments of a single *Porites compressa* colony, which is a dominant reef-building coral in Hawaii, and 5 from separate colonies were used to assess intra- and inter-colony variation in ^1^H-NMR profiles.

Coral metabolite extracts were obtained following methods modified from Gordon et al. [40] to allow for increased extraction times. Solvent choice will significantly influence resulting ^1^H-NMR profiles and consequently the interpretation of an individual’s metabolome. While past studies have used methods combing polar and non-polar solvents to simultaneously extract hydrophilic and hydrophobic metabolites [41], the current application of a 70% methanol/water (v/v; 70% MeOH) solvent system was developed to capture a broad range of the coral’s metabolome without introducing added variation by minimizing extraction steps [40].

All *P. compressa* samples were extracted following extraction method 1. Only inter-colony samples were re-extracted using method 2 to determine if variation in metabolite profiles differed between protocols used in the current study.

#### Extraction Method 1

1 mL of pre-cooled 70% MeOH was added for every 0.7 g of coral to assess technical, intra-, and inter-colony variability. All extracts were sonicated for 15 min and shaken for 24 h at 4°C. To ensure samples were fully extracted, a second solvent volume was added to each coral fragment for an additional 24 h at 4°C. The two resulting extracts were combined and cellular debris removed by centrifugation (4000 rpm for 10 min at 4°C). The supernatant containing the extracted metabolites was removed from the pellet and concentrated using a speed-vacuum concentrator. Extract weights were obtained prior to data acquisition and used to normalize ^1^H-NMR spectra.

#### Extraction method 2

Five inter-colony samples were also extracted by adding 2 mL of 70% MeOH (v/v) for every 0.1 g of coral. Extracts were sonicated for 15 min, mixed on ice for 45 min, and concentrated using a combination of rotary evaporation and lyophilization. Extract weights were obtained prior to data acquisition and used to normalize between ^1^H-NMR spectra.

#### Spiking Experiment

Eighteen fragments from a *P. compressa* colony were collected from a small region (c.a. 135 m^2^) of a fringing reef adjacent to HIMB in December 2011. Fragment volumes were assessed by displacement of deionized (DI) water, which was used to remove excess salt and to determine the amount of solvent to add to each nubbin during metabolite extraction. While the addition of DI water may activate enzymatic pathways resulting in changes in metabolite composition, the identical treatment across all samples allows for comparison of coral metabolomes. Subsequently, fragments were immersed in liquid nitrogen to halt metabolism. To evaluate the capacity of ^1^H-NMR methods to identify differences in concentrations of coral metabolites, 1 M alanine, 1 M glycolic acid, and 1 M glucose were combined and diluted with DI water to prepare 0.1 mM and 10 mM metabolite cocktail spikes. Directly prior to metabolite extraction, 10 µL of the treatment and control (DI water only) cocktails were added to the surface of each whole coral nubbin (n = 6/treatment). Five mL of 70% methanol was added to each fragment for every 1 mL of coral volume (v/v) and metabolites were extracted following method 2 described above.

#### Species Comparison

Replicate fragments (n = 8–9) of *Montipora aequituberculata, Pocillopora damicornis, Porites lobata,* and *Seriatopora hystrix* were collected from Nanwan Bay, Taiwan (ca. 21°56′31″N, 120°44′56″E) in the June of 2011 and transported to the National Museum for Marine Biology and Aquarium (NMMBA), Checheng, Taiwan. These coral species are dominant reef-building corals in Taiwan and represent ecologically distinct taxa [13] with varying morphological and physiological characteristics [42]. Corals were re-fragmented and allowed to recover in a flow-through holding tank (environmental conditions reported Table 1) for two weeks prior to sampling for metabolome analysis. Corals were briefly rinsed with DI water to remove excess salts, immediately immersed in liquid nitrogen, lyophilized, pulverized and transported on dry ice back to the HIMB where they were stored at −80°C. Metabolites were extracted following extraction method 2 described above.

**Table 1:** Flow-through tank conditions prior to sampling of reef-corals at the National Museum for Marine Biology and Aquarium.

Parameter	Mean ± SE
Temperature	27.6±0.03°C
Salinity	33.8±0.02 ppt
Light	107±3.85 µmol photon

*Measurements span the 2-week acclimation period in July 2011.

### NMR Spectroscopy

Coral extracts were reconstituted in 250 µL of deuterium oxide (D_2_O) containing 1 mM of 3-(trimethylsilyl)propionic acid sodium salt (TMSP-*d_4_*) to facilitate comparison of resulting profiles to metabolites in ^1^H-NMR databases. Extracts were briefly sonicated and transferred to a 3 mm NMR tube. ^1^H-NMR profiles were obtained using a 500 MHz Varian Unity Inova spectrometer equipped with a 1M/x-broadband 3 mm probe. Spectra were acquired using a water suppression pulse sequence (PRESAT), consisting of 132 (extraction method 1) or 64 (extraction method 2) transients of 32 K data-points with a relaxation delay of 1 s (extraction method 1) or 3 s (extraction method 2) over a spectral window of 5500 Hz. Resulting spectra were zero-filled to 64 K and multiplied by a line-broadening factor of 0.5 Hz prior to Fourier transformation. Spectra were imported in to MestreNova (Mestrelabs version 7.1.2), where spectral baselines were adjusted using Whittiker smoothing and normalized to the total area. Three alignments were created to compare ^1^H-NMR (1) profile variability, (2) spectra spiked with the metabolite cocktails, and (3) fingerprints among coral species. All alignments were reduced to ASCII files. Variables corresponding to the residual water impurity (4.48–4.92 ppm) and an observed acetone contaminant (2.22–2.27 ppm) were removed. Alignments were imported into Metabolink (http://metabolink.knoesis.org), where a dynamic adaptive binning routine was used to identify each peak as a separate variable [43]. For each alignment, bins were found between 0.5 to 10 ppm. R statistical environment (version 3.0.0, R Development Core Team 2013, http://www.R-project.org) was used for all further analysis, including normalizing spectral intensities to extract weights for comparison across samples.

### Data Analysis

#### Univariate Analyses

To investigate variability in ^1^H-NMR spectra, relative standard deviations (% RSD = mean/standard deviation × 100; reported as median % RSD) were calculated across variables for each group [44]. A Kruskal-Wallis analysis was applied to compare values among technical, intra-, and inter-colony spectra from *Porites compressa*.

In the spiking experiment, Chenomx NMR Suite 7.6 (Chenomx, Inc., Edmonton, Alberta, Canada) was used to identify and quantify ^1^H-NMR signals resulting from the metabolite spikes. A Kruskal-Wallis test was used to statistically compared metabolite concentrations of alanine, glucose, and glycolic acid among treatment groups.

#### Multivariate analysis

All variables from the three alignments were scaled to unity and mean centered prior to multivariate analysis. Both principal component analysis (PCA) and orthogonal partial least squares-discriminate analyses (OPLS-DA) were used to investigate patterns in variables arising from ^1^H-NMR spectra.

PCA is an unsupervised pattern recognition tool that seeks to explain the maximum amount of variation inherent to a multi-dimensional dataset. As such, PCA was applied to investigate patterns between ^1^H-NMR profiles. Additionally, PCA was used to screen for outlying samples. If spectra fell outside a 99% confidence interval and upon further inspection it was determined that NMR shims influenced resulting peak shapes and line widths, spectra were excluded from subsequently analyses (e.g., Fig. S1).

OPLS-DA is a supervised pattern recognition technique that aims to find the maximum separation between *a priori* groups [45], [46]. OPLS-DA was applied to discriminate between ^1^H-NMR profiles arising from (1) spiking treatments and (2) among coral species (for source code see http://birg.cs.cofc.edu/index.php/O-PLS). Model strength was assessed using both R^2^ and Q^2^ metrics. R^2^ values report the total amount of variance explained by the model in both the ^1^H-NMR data (R^2^X) and independent variables (R^2^Y; e.g., spiking treatment or species identity). Q^2^ reports model accuracy and is calculated by 10-fold cross validation. The resulting Q^2^ statistic was compared to a null distribution to test model significance (p<0.05). OPLS-DA is advantageous over analogous methods (e.g., partial least squares-discriminate analysis) because it looks to partition between-group variation (t) in ^1^H-NMR profiles from within-group variation (t-orthogonal), which enhances the interpretability of the resulting model [46].

#### Implementing the n-group OPLS-DA model

OPLS-DA typically is employed to discriminate between two treatment groups [46]. However, when there are more than three groups, the resulting model is influenced by group order. To facilitate comparing metabolite fingerprints among spiking treatments and reef-building corals, we developed an iterative strategy based on the magnitude of Q^2^ to determine group ordering in the model. The algorithm finds the maximum Q^2^ value between the two groups with the largest separation along the t-axis. Additional groups are inserted into the model based on the magnitude of Q^2^. The overall Q^2^ value is determined from model projections after allowing groups to cluster together based on profile similarities. This strategy allows the OPLS-DA model to dictate where added groups should reside with respect to those present. Furthermore, it provides information describing the similarity between groups (i.e., groups with similar profiles will have similar t values and be plotted closer together). Finally, the OPLS-DA algorithm calculates coefficients describing the contribution of each variable to the model.

#### Variable Selection and Metabolite Identification

To determine which variables drive separation in metabolite composition among coral species, variable coefficients from the OPLS-DA model were compared to their null distributions. Null distributions were calculated by refitting the OPLS-DA model to the data, in which each variable is independently and randomly permuted to remove correlation structure. The actual coefficients were compared to their null distributions and variables in the tails (α = 0.01) were determined to significantly contribute to the model.

To facilitate identification of the metabolites driving separation between species, a statistical total correlation spectroscopy analysis (STOSCY) in the R package MUMA [47] was used to determine strong correlations between ^1^H-NMR variables. Highly correlated variables (r^2^>0.9) were assumed to originate from the same compound [48]. Metabolites were assigned by matching peak positions and patterns to Chenomx 500 MHz spectral libraries.

## Results

### Technical, Intra-, and Inter-Colony Variability

**Figure 1:**
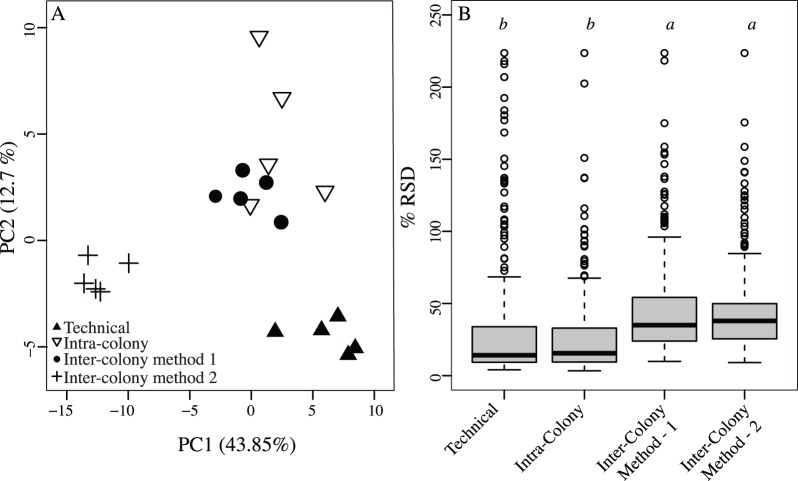
^1^H-NMR profiles of *Porites compressa* are reproducible within and between coral colonies. (A) PCA comparing *Porites compressa* ^1^H-NMR metabolite profiles between technical, intra-colony and inter-colony samples. Profiles from inter-colony *P. compressa* samples were obtained using two extraction methods: method 1 and method 2 (B) Boxplots of percent relative standard deviation (% RSD) scores across ^1^H-NMR variables comparing technical, intra- and inter-colony variability. The median is indicated (black bar) along with the quartile ranges and outlying values (open circles). Letters denote Kruskal-Wallis test results (p<0.001). Groups connected by the same letter are not significantly different.

Variability in *Porites compressa* ^1^H-NMR spectra was explored over 284 spectral bins (variables) describing metabolite profiles. PCA revealed close clustering of samples among technical and intra-colony replicates, while inter-colony samples were slightly more dispersed (Fig. 1A). A Kruskal-Wallis comparison of relative standard deviation (RSD) scores quantifies these visual patterns, where technical (median 14.2%) and intra-colony (15.2%) scores were not statistically different from one another, but both were significantly lower than inter-colony scores (p<0.001, 35% and 38%, Fig. 1B). While the two extraction methods formed separate groups (Fig. 1A) in the PCA, RSD scores were not significantly different following the Kruskal-Wallis analysis (35% vs. 38%). Thus, while metabolite composition may vary between protocols, variability in ^1^H-NMR profiles is similar.

### Spiking Experiment

**Figure 2:**
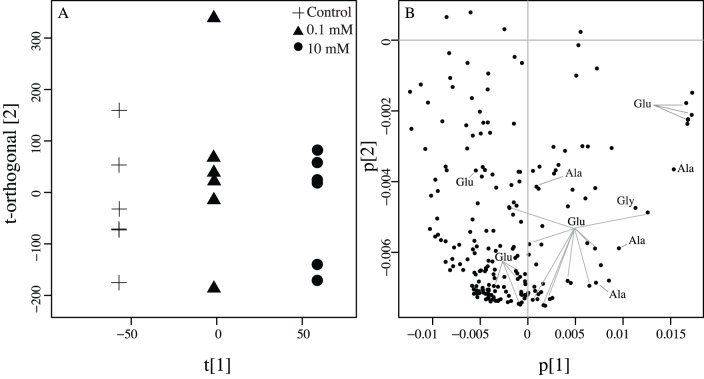
Separation in metabolite profiles after experimental addition of alanine, glucose, and glycolic acid. (A) OPLS-DA model comparing the control, 0.1 mM, and 10 mM metabolite spiking treatments. Separation within and between treatments is represented by the t-orthogonal- and t-axis, respectively. Model statistics are reported (Table 2). (B) Corresponding loading plot showing ^1^H-NMR bin coefficients. Bins arising from each spiking compound are indicated. Ala = alanine, Glu = glucose, Gly = glycolic acid.

^1^H-NMR profiles arising from the spiking experiment were binned into 208 variables. The resulting OPLS-DA model revealed clear separation of metabolite profiles (Fig. 2, Table 2) among treatments (p<0.01, R^2^X = 0.09, R^2^Y = 0.99, Q^2^ = 0.45). Of the three spiked compounds, alanine and glucose, but not glycolic acid, were successfully identified and quantified in control spectra. All three metabolites were detected in both the 0.1 mM and 10 mM spiking treatments (Fig. S2). However, when comparing mean concentrations across groups for alanine, glucose, and glycolic acid using a Kruskal-Wallis ANOVA, only the 10 mM treatment was significantly different from the control and 0.1 mM groups (p<0.05, Table 3).

**Table 2:** OPLS-DA Model Results.

Model	R^2^X*	R^2^Y*	Q^2^ *	p-value
Spiking Experiment	0.09	0.99	0.48	<0.01
Species Comparison- All Species	0.25	0.95	0.89	<0.01
*M. aequituberculata* and *P. damicornis*	0.25	0.80	0.66	<0.01

*R^2^X and R^2^Y represent the goodness of fit between the X (metabolite data) and Y (predictor values) matrices. Q^2^ assesses the accuracy and predictability of the model. A Q^2^ value close to 1.0 represents a more predictive model.

**Table 3:** Kruskal-Wallis test results comparing spiking treatments.

Metabolite	Treatment	Mean ± SE(mM g^−1^ extract weight)	Chi-Square	p-value	Kruskal-WallisGroupings
Alanine	Control	6.61±3.20	11.415	0.0033	b
	0. mM	5.12±1.09			b
	10 mM	145.83±37.77			a
Glucose	Control	9.51±4.54	11.368	0.0038	b
	0. mM	3.54±0.45			b
	10 mM	87.79±22.12			a
Glycolate	Control	ND*	15.725	0.00038	ND
	0.1 mM	3.12±1.45			b
	10 mM	80.09±27.66			a

*ND = Not Detected.

### Species Comparison

**Figure 3:**
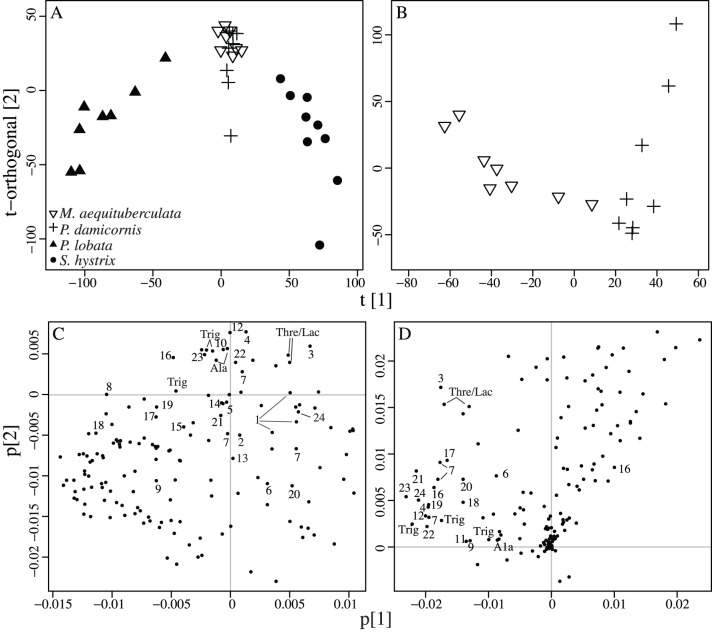
Reef-building corals have species-specific ^1^H-NMR profiles. OPLS-DA models comparing ^1^H-NMR profiles from (A) *Montipora aequituberculata, Pocillopora damicornis*, *Porites lobata* and *Seriatopora hystrix* and (B) between *M. aequituberculata* and *P. damicornis* only. Separation within and between species is represented by the t-orthogonal- and t-axis, respectively. Model statistics are reported (Table 2). (C and D) Corresponding loading plots showing ^1^H-NMR-bin coefficients. Variables driving separation in the 4-species OPLS-DA model (A) are identified with numbers corresponding to unknowns (Table 4). Only significant variables are indicated for each model. Ala = Alanine, Trig = Trigonelline, Thre/Lac = Threonine/Lactate.

Dynamic adaptive binning identified 152 variables describing ^1^H-NMR fingerprints arising from *Montipora aequituberculata, Pocillopora damicornis, Porites lobata,* and *Seriatopora hystrix* aligned spectra. The OPLS-DA model revealed clear significant discrimination between metabolite profiles originating from *P. lobata* and *S. hystrix* corals. However, OPLS-DA was unable to separate profiles from *M. aequituberculata* and *P. damicornis* (P<0.01, R^2^X = 0.25, R^2^Y = 0.95, Q^2^ = 0.89; Fig. 3A, Table 2). Consequently, to determine if OPLS-DA could significantly discriminate between metabolite profiles from all coral species analyzed, all possible combinations were modeled (Fig. S3, Table S1). The OPLS-DA comparison of profiles from *M. aequituberculata* and *P. damicornis* revealed significant separation in metabolite profiles (p<0.01, R^2^X = 0.25, R^2^Y = 0.79, Q^2^ = 0.75, Fig. 2B, Table 2).

From the OPLS-DA model comparing all 4 species, 35 bins were found to drive separation among coral profiles. STOCSY categorized these variables into 27 individual compounds (Table 4), of which only three (alanine, trigonelline, threonine, and/or lactate) were matched to Chenomx spectral libraries. The remaining unidentified compounds contain proton signals matching spectral locations of lipids, amino acids, organic acids, and carbohydrates.

**Table 4:** Variables driving separation in metabolite fingerprints among coral species.

Bin Center	Peak Pattern*	Annotation	Compound Class
0.938	m	Unknown 1	Branch-chained amino acids
0.983	m		
1.011	m		
1.090	m	Unknown 2	Branch-chained amino acids
1.306		Unknown 3	Aliphatic
1.327	d	Threonine/Lactate	Organic Acid
1.337			
1.390	m	Unknown 4	Lipid
1.454	d	Alanine	
1.496			
1.740	m	Unknown 5	Aliphatic
2.031	m	Unknown 6	Aliphatic
2.051	m	Unknown 7	Aliphatic
2.072			
2.086			
2.670	m	Unknown 8	Aliphatic
2.707	t	Unknown 9	Aliphatic
2.779	s	Unknown 10	Aliphatic
2.870	m	Unknown 11	Aliphatic
2.986	s	Unknown 12	Aliphatic
3.031	m	Unknown 13	Aliphatic
3.046	m	Unknown 14	Aliphatic
3.160	s	Unknown 15	Aliphatic
3.380	m	Unknown 16	Carbohydrates
3.555	m	Unknown 17	Carbohydrates
3.566	m	Unknown 18	Carbohydrates
3.583	m	Unknown 19	Carbohydrates
3.649	s	Unknown 20	Carbohydrates
3.985	m	Unknown 21	Carbohydrates
4.031	s	Unknown 22	Carbohydrates
4.321	m	Unknown 23	Carbohydrates
5.152	d	Unknown 24	Carbohydrates
4.446	s	Trigonelline	
8.842	m		
9.135	m		

*Peak patterns: s-singlet, d-doublet, m-multiplet.

## Discussion

### ^1^H-NMR metabolomic profiling is reproducible

^1^H-NMR metabolite profiles obtained from reef-building corals were highly reproducible. Intra-colony variation in ^1^H-NMR spectra from *P. compressa* was low and equal to that of technical replicates. As expected, variation in metabolite fingerprints is higher among different coral colonies. Together, these results indicate that while metabolite composition is relatively homogeneous within a coral colony, genotypic differences among colonies elevate variability in metabolite profiles. Notably, the variability in metabolite profiles described here are consistent with reports [44] for fish, marine invertebrates, and mammals, where relative standard deviations (RSD) across ^1^H-NMR bins are lower across technical replicates (median RSD range 1.6–20.6%) and increase with biological replication (median RSD range 7.2–58.4%).

Low technical variability is a critical attribute for any method, including metabolite profiling, that is applied to uncover patterns associated with shifts in performance or metabolism in response to ecological drivers. High levels of variation cloud researchers’ ability to detect significant shifts in metabolic performance. Because ^1^H-NMR techniques tend to be highly reproducible [49], they have become popular in monitoring organism health in response to the environment. For instance, ^1^H-NMR methods have uncovered patterns in metabolite profiles across a range of organisms in response to pollution [50]–[52], shifts in temperature regimes [53], [54] and increases in ocean acidification [55], [56]. We add to this body of literature by demonstrating that ^1^H-NMR metabolite profiling methods are reproducible for reef-building corals, indicating that this approach is likely to have high value in monitoring metabolic state either in field or laboratory experiments.

### Complete profiles distinguish small differences in ^1^H-NMR profiles

The OPLS-DA model discriminated between ^1^H-NMR spectra measured from the three spiking treatment groups (control, 0.1 mM and 10 mM), leading to significant separation in metabolite profiles (Fig. 2). However, after Chenomx identified and quantified signals from the spiking compounds (alanine, glucose, and glycolic acid), a Kruskal-Wallis test only detected significant differences in the 10 mM treatment group in comparison to the others (Table 3). These results suggest that detecting small differences in individual metabolite concentrations (i.e., between the control and 0.1 mM treatments) may be constrained by dynamic signals within the coral metabolome or by background noise in ^1^H-NMR spectra. It is clear that the majority of the ^1^H signals arising from alanine, glucose and glycolic acid fall in regions of high peak overlap in coral ^1^H-NMR spectra (Fig. S2 and Fig. S4). This limits the ability to detect small differences in metabolite concentrations. For instance, a recent targeted application of ^1^H-NMR spectroscopy detected significant differences in dimethylsulphoniopropionate and acrylate, both of which resonate in regions of low spectral complexity, as low as 1.4 nmol/mm^2^ between corals exposed to ambient and high temperature conditions [39]. These compounds did not contribute to separation in metabolite profiles among spiking treatments or coral taxa investigated here, which is expected given that they naturally occur in similar concentrations across individuals [38]. While targeted studies using ^1^H-NMR techniques are informative towards describing and quantifying known metabolites, shifts in coral metabolomes may occur inside regions of high peak overlap. Furthermore, by comparing spectra with multivariate techniques, we can identify combined signals that better resolve differences in metabolite concentrations. Taken together, the current data suggest multivariate techniques enhance the capacity to identify small fluctuations in coral ^1^H-NMR profiles in comparison to univariate methods. Consequently, when using ^1^H-NMR techniques to investigate coral metabolomes analyzing complete spectra may be more informative than comparing changes in individual compounds.

### ^1^H-NMR profiling methods can detect species-specific signatures

Our ^1^H-NMR profiling approach and OPLS-DA indicate that different reef-building coral species have distinct metabolite profiles. While the separation in the OPLS-DA model suggests that spectra from *Porites lobata* and *Seriatopora hystrix* are very different, it has limited the capacity to discriminate between *Pocillopora damicornis* and *Montipora aequituberculata* profiles (Fig. 3A). However, OPLS-DA can discriminate between *P. damicornis* and *M. aequituberculata* when these two species are modeled independently of the others (Fig. 3B). These data suggest that there are distinct elements in coral metabolite profiles that are both similar between *P. damicornis* and *M. aequituberculata*, and that differentiate the four species.

Using the variable selection algorithm and a STOCSY analysis [48], 27 compounds from a broad range of metabolite classes including lipids, amino acids, organic acids, and carbohydrates contribute to the separation in coral metabolite profiles. However, of these 27 compounds, only a few were matched to Chenomx database entries and include trigonelline, alanine, several branch chained amino acids (e.g., valine, isoleucine and leucine), and organic acids (threonine or lactate). Our work highlights the challenges associated with metabolite identification using ^1^H-NMR metabolomics. NMR instrumentation is less sensitive than mass spectrometry. Overlapping signals, variation in sample pH, ionic strength, temperature and acquisition conditions can obscure accurate database assignments. Past studies have typically identified between 2 and 15 compounds that separate ^1^H-NMR profiles in non-model species such as *Mytilus edulis* or *Carcinus maenas* exposed to disturbance (e.g., copper exposure or ocean acidification [52], [56]). In contrast, ^1^H-NMR-based metabolomics have identified upwards of 30 metabolites driving differences in the metabolism of model organisms (e.g., impacts of *Mycobacterium tuberculosis* infections in mice [57]). Lack of taxa-specific metabolite databases reduces the capacity to identify small compounds in non-model species. To overcome these challenges, research is focused on developing analytical (e.g., cyroprobes, increases in magnet strength, 2D-NMR techniques; reviewed by [58], [59]), bioinformatics [60]–[64] and databases tools [65] to facilitate matching ^1^H-NMR profiles to known metabolites.

Despite limitations associated with identifying metabolites in ^1^H-NMR spectra, the patterns in metabolite composition observed in coral profiles are intriguing. The four species investigated represent physiologically distinct taxa [42] that respond differently to environmental disturbances [13]. ^1^H-NMR profiles are indicative of metabolite composition and consequently the activity of metabolic pathways. The observed variation among species rationalizes further exploration of the metabolome to describe differential responses of corals to the environment and anthropogenic stress.

## Conclusions

Our results illustrate the capacity of ^1^H-NMR metabolomics to describe, compare and assess coral metabolomes. Future application of these methods, coupled with rigorous ecological monitoring [66], may enable researchers to document shifts in metabolite composition across time and environmental conditions. As global climate change and other local stressors continue to threaten reefs, ^1^H-NMR tools may aid researchers in the rapid assessment of coral reef metabolic states.

## Supporting Information

Figure S1 **PCA identifies two outlying metabolite profiles when comparing spectra between reef-building coral species**. PCA comparing metabolite profiles between *Montipora aequituberculata, Pocillopora damicornis*, *Porites. lobata* and *Seriatopora hystrix* (A) with and (B) without outlying samples. Ellipse represents a 99% confidence interval.
(EPS)

Figure S2 **Complex profiles result in high signal overlap.** Expanded regions of ^1^H-NMR spectra showing the location of ^1^H-resonances for alanine, glucose, and glycolic acid. Turquoise = control, pink = 0.1 mM, dark blue = 10 mM.
(EPS)

Figure S3 **All possible OPLS-DA models demonstrate reef-building corals have species-specific ^1^H-NMR profiles.** (A) 4-species model, (B–E) 3-species models, and (F–K) pair-wise species comparisons of metabolite profiles from *Montipora aequituberculata, Pocillopora damicornis*, *Porites lobata* and *Seriatopora hystrix*. Model statistics are reported (Table S1).
(EPS)

Figure S4 ***P. compressa*** **profiles are visually similar, except in regions of spiking compounds.** Representative ^1^H-NMR spectra from the metabolite spiking experiment of the 10 mM (A), 0.1 mM (B) and control (C) treatments.
(EPS)

Table S1 **All possible OPLS-DA models comparing reef coral ^1^H-NMR profiles.**
(DOCX)
