# Supplementary material for: Application of 1H-NMR Metabolomic Profiling for Reef-Building Corals
Source: PLoS One. 2014 Oct 29;9(10):e111274. doi: 10.1371/journal.pone.0111274 (PMC4213140; doi:10.1371/journal.pone.0111274)
Supplement: Table S1 — All possible OPLS-DA models comparing reef coral 1H-NMR profiles. (DOCX) [file pone.0111274.s005.docx]

**Table S1. All possible OPLS-DA models comparing reef coral ^1^H-NMR profiles.**

| **Model Letter*** | **Model** | **R^2^X**** | **R^2^Y**** | **Q^2^**** | **P-Value** |
| --- | --- | --- | --- | --- | --- |
| A | All Species | 0.25 | 0.25 | 0.88 | < 0.01 |
| B | *P .lobata, P. damicornis,*  *M. aequituberculata* | 0.36 | 0.36 | 0.81 | < 0.01 |
| C | *P. lobata, P. damicornis*, *S. hystrix* | 0.31 | 0.31 | 0.90 | < 0.01 |
| D | *P. damicornis,* *M. aequituberculata,*  *S. hystrix* | 0.16 | 0.16 | 0.78 | < 0.01 |
| E | *P. lobata*, *M. aequituberculata*,  *S. hystrix* | 0.32 | 0.32 | 0.87 | < 0.01 |
| F | *P. lobata, P. damicornis* | 0.43 | 0.90 | 0.84 | < 0.01 |
| G | *P. lobata*, *M. aequituberculata* | 0.45 | 0.93 | 0.81 | < 0.01 |
| H | *P. lobata,* *S. hystrix* | 0.43 | 0.94 | 0.91 | < 0.01 |
| I | *P. damicornis*, *M. aequituberculata* | 0.25 | 0.80 | 0.66 | < 0.01 |
| J | *P. damicornis,* *S. hystrix* | 0.24 | 0.90 | 0.86 | < 0.01 |
| K | *S. hystrix*, *M. aequituberculata* | 0.27 | 0.92 | 0.84 | < 0.01 |

*Model letters correspond to panels in Fig S1

**R^2^X and R^2^Y represent the goodness of fit between the X (metabolite data) and Y (predictor values) matrices. Q^2^ assesses the accuracy and predictability of the model. A Q^2^ value close to 1.0 represents a more predictive model
